# Potential suitable habitat of *Eleusine coracana* (L) gaertn (Finger millet) under the climate change scenarios in Nepal

**DOI:** 10.1186/s12898-020-00287-6

**Published:** 2020-04-06

**Authors:** Dol Raj Luitel, Mohan Siwakoti, Mohan D. Joshi, Muniappan Rangaswami, Pramod K. Jha

**Affiliations:** 1 Central Department of Botany, Tribhuvan University, Kirtipur, Kathmandu, Nepal; 2 Department of Environment, Ministry of Forests and Environment, Kathmandu, Nepal; 3 Department of Plant Resources, Ministry of Forests and Environment, Kathmandu, Nepal; 4 IPM-IL, Virginia Tech, Blacksburg, USA

**Keywords:** Climate change, Finger millet, Habitat suitability, Maxent model

## Abstract

**Background:**

Finger millet is the fourth major crop in Nepal and is cultivated in a traditional integrated subsistence system. Timely rain and appropriate temperature predominately affects crop distribution and yield. Climate change is evident in Nepal and it is imperative to understand how it affects habitat suitability of finger millet. Main objective of this study was to map the current suitable habitat and predicting the potential changes in the future under different climate scenarios in Nepal. Habitat mapping is important for maximizing production and minimizing the loss of local landraces.

**Results:**

Maxent model was used in this study to quantify the current suitable habitat and changes in the future habitat suitability of finger millet, based on representative concentration pathways (RCP) (RCP 2.6, 4.5, 6.0 and 8.5) in two different time periods (2050 and 2070AD) using climatic predictive variables and species localities. The model shows that 39.7% (58512.71 km^2^) area of Nepal is highly suitable for finger millet, with cultivation mostly between 96 and 2300 m above sea level. Eastern and central parts of Nepal have more suitable areas than western parts. Our research clearly shows that the future climatic suitable area of finger millet would shrink by 4.3 to 8.9% in 2050 and 8.9–10.5% under different RCPs by 2070.

**Conclusion:**

Finger millet is mostly cultivated in mid-hill terraces. The substantial increase in temperature due to climate change may be one reason for decrease in habitat suitability of finger millet. This situation would further threat loss of local landraces of finger millet in the future. The findings can help in planning and policy framing for climate resilient smart agriculture practice.

## Background

Nepal, having an area of 147,181 km^2^, lies on the southern slope of the Central Himalayas. It has a wide range of variation in physiographic, topographic, climatic and edaphic conditions, and has tropical to tundra climate within a narrow range of 185 km North–South. Rice, wheat, maize, finger millet and buckwheat are the major cereal crops. Agriculture is the major source of economy, contributing 32% of total GDP and employing 63% of the population. The rural population of Nepal depends primarily on traditional subsistence, rain-fed agricultural practice, which is climate dependent [1]. Finger millet [*Eleusine coracana* (L.) Geartn, family: Poaceae], the fourth major crop in Nepal, occupies an important role in Nepalese agriculture, especially in mountainous remote areas [2]. It is considered an under-exploited, poor person’s, neglected crop, cultivated mostly in mid-hills and mountainous regions [3, 4].

Finger millet is the fourth cereal in term of area and production after rice, wheat and maize with total cultivation area was 265,496 ha in 2007 and 263,596 ha in 2017.The national production of finger millet was 291,098t in 2007and increased to 306,704t in 2017 [2]. The increase in production of this crop cannot fulfill the national demand so it is being imported from neighboring countries. The import of finger millet was 1217t in 2017 [2]. It is evident that both the national demands as well as yield are continuously increasing. Finger millet’s grain is rich in protein, dietary fiber and minerals especially calcium, iron, zinc and phosphorus as compared to other cereals [5, 6] and play an important role in food and nutrition security in areas where they are grown [7].

Future projections based on four representative concentration pathways (RCPs) (2.6, 4.5, 6.0 and 8.5) show that the earth’s temperature will increase by 2.6–3.8 °C by 2100AD. In Nepal, the mean annual temperature is projected to increase by 1.4 °C by 2030, 2.8° C by 2060, and 4.7 °C by 2090. Based on the Regional Circulation Model (ReCM), the total precipitation and its intensity may increase by 15–20% during the summer currently [8]. The total precipitation is projected to increase by 6% and 12% by 2050 and 2090, respectively [9]. However, precipitation projection from observed historical trends shows a decrease in post-monsoon rainfall [10] with more erratic, heavy, and unpredictable rain within shorter periods, potentially leading to extreme drought [11]. Similarly, the annual precipitation of Nepal has increased by 13 mm and days of rainfall have decreased by 0.8 days per year since 1990 [12]. These changing patterns of climatic variables are expected to severely affect crop production, livelihoods, and food security in Nepal [13].

Species distribution modelling (SDM) is one of the simple and quick tools for identifying the climatic changes and projections of climatic impacts on species and can be used to match adaptation policies and practices [14, 15]. Understanding exact suitable area and predicting future suitable area of a crop is essential for understanding further expansion or shrinkage of cultivation areas that may open new avenues for rescuing neglected local landraces. In Nepal, studies on the effects of climatic variables on modeling for habitat suitability of minor crop species are virtually absent. This work not only fills that knowledge gap, but also opens new avenues for analyzing the habitat of crop species in response to different environmental variables and predicts the habitat suitability by using the machine learning software Maxent [16].

## Results

### Contribution of predictor variables and model accuracy

**Fig. 1:**
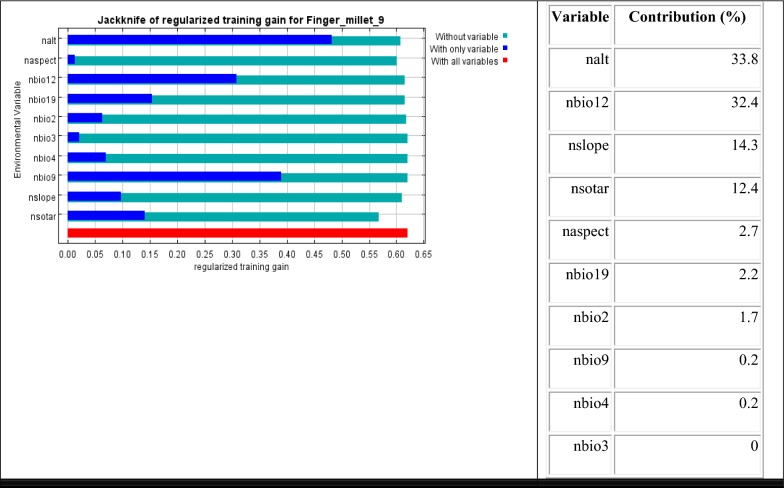
The results of the Jackknife test on variables’ contribution to modelling finger millet habitat suitability. The regularized training gain describes how much better the Maxent distribution fits the presence data compared to a uniform distribution. The dark blue bars indicate the gain from using each variable in isolation, the light blue bars indicate the gain lost by removing the single variable from the full model, and the red bar indicates the gain using all of the variables. The contribution of individual variables is presented in right side within the figure

From the Jackknife analysis, among the nine bioclimatic variables used for modeling altitude (nalt), annual rainfall (nbio12), slope of the region (nslope), and soil (nsotar)together contributed about 93% in predicting the suitable habitat of finger millet (Fig. 1). Altitude (nalt) had the highest contribution of 33.8%, followed by annual rainfall (nbio12) with 32.4% contribution to the model. The mean temperature of the driest quarter (nbio9), aspect (naspect), mean diurnal range (nbio2), and temperature seasonality (nbio4)variables showed the least contributions, but the isothermality (nbio3) showed no contribution (Fig. 1).

Overall accuracy was high (> 0.80, which refers to 80% accuracy) for predictions under present and future time periods by AUC. The model performances evaluated by different statistic score/matrices are given in Table 1. Validity of the model for current habitat suitability of finger millet was strong with AUC = 0.832, indicating that the variables used in modeling described well to determine the habitat suitability of finger millet in Nepal. The AUC, prediction accuracy of the model used for analyzing the distribution under present and future time period of finger millet, ranged from 0.806 to 0.845, TSS between 0.576 and 667, and the Kappa between 0.601 and 0.631.

**Table 1:** Prediction accuracy of habitat suitability in distribution modeling of finger millet

Model Evaluator	Current	RCP2.6_ 2050	RCP 2.6 _ 2070	RCP4.5_ 2050	RCP4.5_ 2070	RCP6.0_ 2050	RCP6.0_ 2070	RCP8.5_ 2050	RCP8.5_ 2070
AUC	0.832	0.833	0.841	0.845	0.841	0.838	0.838	0.806	0.838
Sensitivity	0.866	0.868	0.865	0.852	0.904	0.852	0.847	0.863	0.863
Specificity	0.712	0.799	0.704	0.773	0.741	0.758	0.7150	0721	0.742
TSS	0.576	0.667	0.617	0.626	0.645	0.610	0.608	0.584	0.605
Kohan’s Kappa	0.621	0.607	0.606	0.631	0.601	0.616	0.622	0.616	0.626

### Distribution of suitable habitat

**Fig. 2:**
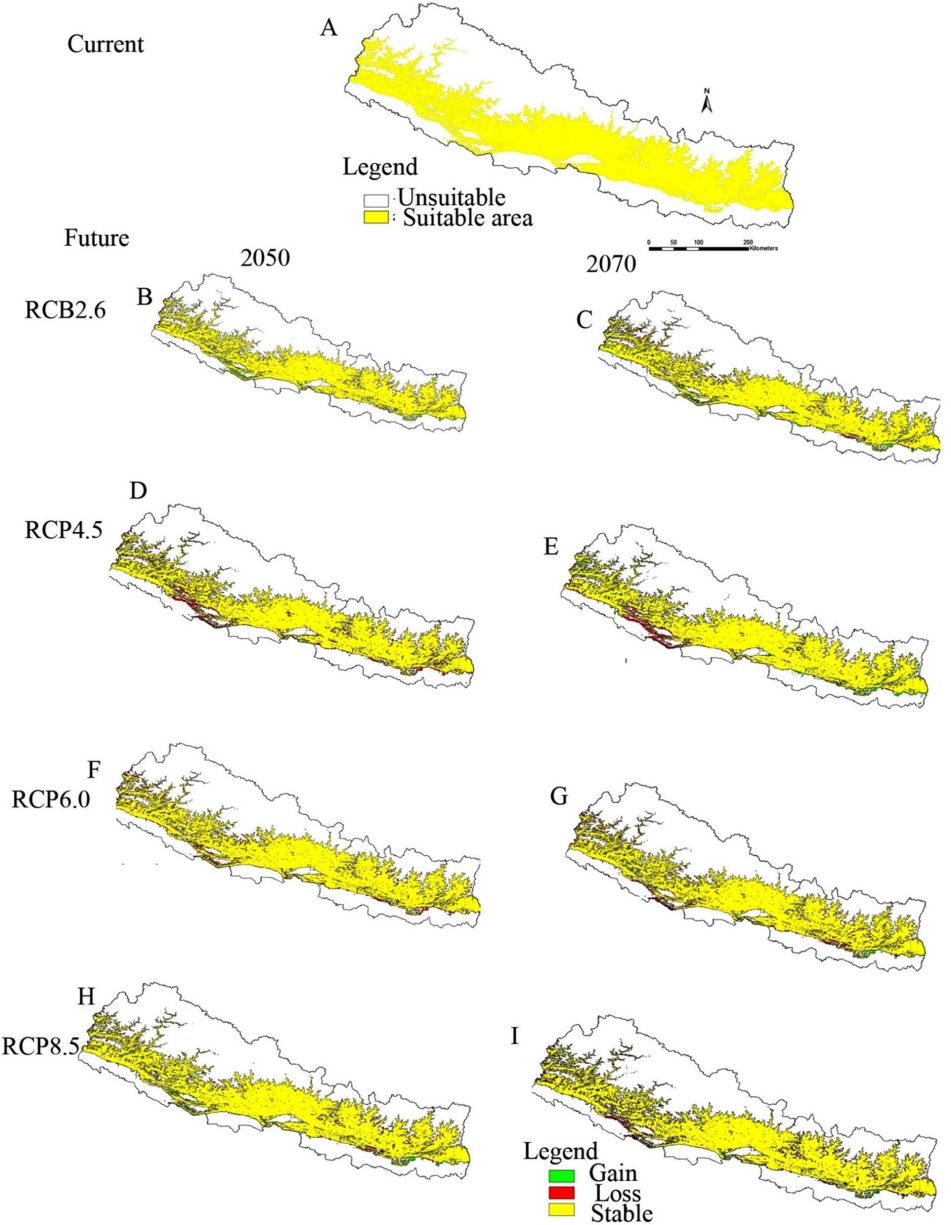
Predicted habitat suitability of finger millet in Nepal (**a** = current, **b**–**i** = future).Future scenarios show changes in habitat suitability of finger millet crop in Nepal in different RCP’s (Red region represents the area loss, green represents the gain area, and yellow represents stable or no change area with respect to current situations

Under present climatic scenario, the overall distribution of most suitable habitat of finger millet lies between 500 and 1500 m elevation above the sea level (asl) although it occurs between the elevation range between 96 and 2300 m asl (Fig. 2a and Table 3). In the current scenario, total suitable habitat for finger millet cultivation was 39.7% (58512.71 km^2^) (Table 2). Physiographically, the predicted potential habitat suitability suggested that the most suitable areas for finger millet are the Siwalik, mid-hill, and lower part of mountain region of Nepal (Fig. 2a). Similarly, the central and eastern part of the country is more suitable than the western parts.

**Table 2:** Habitat stability, gain and loss area Km^2^ of finger millet under different RCPs in 2050 and 2070

Climatic scenarios	Stable area	Gain area	Loss area	Net gain or loss area	Significance
Current	58512.71(100)				
Future					
RCP 2.6_year 2050	56082.51(95.8)	3401.74(6.1)	2436.77(4.3)	+ 964.97(1.8)	Yes
RCP 2.6_year 2070	56599.69(96.7)	3197.33(5.6)	5133.6(9.1)	− 1936.27(3.5)	Yes
RCP 4.5_year 2050	56018.34(95.7)	2248.46(4.1)	2511.68(4.5)	− 263.22(0.4)	Yes
RCP 4.5_year 2070	55716.17(95.2)	2120.07(3.8)	4932.29(8.9)	− 2812.22(5.1)	Yes
RCP 6.0_year 2050	55716.17(95.2)	2120.07(3.8)	4932.29(8.9)	− 2812.22(5.1)	Yes
RCP 6.0_year 2070	54436.83(93.1)	1595.15(2.9)	5689.2(10.5)	− 4094.05(7.6)	Yes
RCP 8.5_year 2050	56357.93(96.3)	2594.41(4.6)	2166.39(3.9)	+ 428.02(0.7)	Yes
RCP 8.5_year 2070	55184.56(94.3)	2248.46(4.1)	5590.8(10.2)	− 3342.34(6.1)	Yes

Figure in parentheses is percentage of area

### Future distribution suitability scenarios (stable, loss and gain area)

**Fig. 3:**
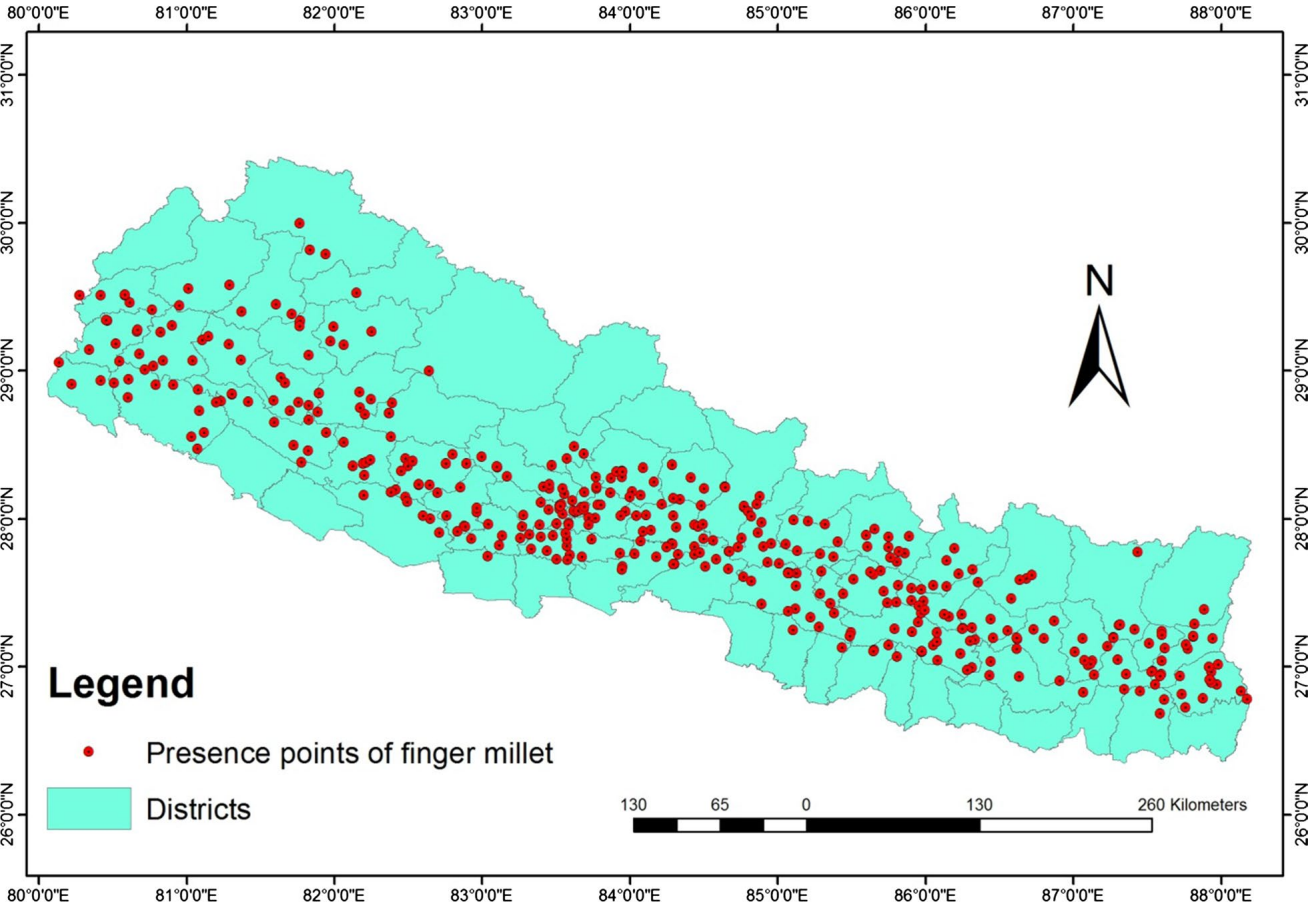
Study area showing the presence points used in modeling of finger millet in Nepal

The distribution of suitable area of finger millet under current and future conditions is presented in Fig. 3. In the future distribution of finger millet, the stable, gain, and loss area are presented in solar yellow, green, and red color, respectively, for no change, increase, and loss in each figure (Fig. 2b–i).

By undertaking visual inspection of the model prediction based on current climatic conditions and occurrence records, it is clear that the western part of the country is comparatively less suitable than eastern and central parts. The future distribution reveals the shift of habitat towards the northeast but reduces the total suitable area in the year 2050 and 2070 as compared to the current situation (Fig. 2).

Our analysis shows that by 2050 there will be decrease in suitable habitat by 4.2%, 4.3%, 4.8% and 3.8% in RCP’s 2.6, 4.5, 6.0 and 8.5, respectively (Table 2). The total loss area in different RCP’s analysis shown by 2050 ranges from 3.9 to 8.9% over the current suitable habitat area and 8.9–10.5% area will be lost by 2070. Similarly, the suitable habitat will gain by 6.1, 4.1, 3.8 and 4.6% in RCP’s 2.6, 4.5, 6.0 and 8.5, respectively, by 2050 and by 5.6, 3.8, 2.9 and 4.1%, respectively, by 2070 (Table 2, Fig. 2 b–i).

### Changes in potential suitable area under climate change scenarios

The predicted suitable area for finger millet under the RCP2.6 climate change scenario would decrease by 3.5% under 2.6 RCP by 2050. Similarly, the net suitable area for finger millet would be lostby 0.4% and 5.1% in RCP 4.5 and 6.0 by 2050, respectively. However, 0.7% area would be gained under extreme conditions of climate i.e. 8.5 RCP by 2050 (Table 2, Fig. 2).The net suitable area would continue to be lost by 3.5 to 7.6% under different RCPs by 2070.

By 2050, in RCP’s 2.6, the major area will shrink in far western Nepal, specifically in Mahakali and Karnali regions in west, and some areas like Sagarmatha and Koshi regions in the east. There may be addition of some areas in the mid-western region of Nepal (Fig. 2b) and there is almost a similar trend for 2070 in 2.6 RCPs (Fig. 3c). In RCP’s 4.5, for both year 2050 and 2070, the major part of suitable area for finger millet will be lost in mid and far regions (Fig. 2d, e). In RCP’s 6.0, the suitable area will shrink throughout the country especially in upper mountain and lower parts of mid-hills, with the exception of some of the areas (Fig. 2f, g) for both 2050 and 2070. There will almost be a similar pattern followed for RCPs 8.5 for 2050 and 2070 (Fig. 2h, i).

At present, the most suitable habitat for finger millet is between 96 and 2300 m asl in Nepal, but is more concentrated in eastern and central Nepal. However, moderate suitable (probability of suitability in between 25 and 50%) area ranged from 75 to 3034 m. In the future, most suitable habitat will shrink from 2300 m to 2151 m in RCP 4.5 in year 2050 and further shrinkage to 2097 m asl in RCP 8.5 in year 2050 in Nepal. Similarly, there will be a decrease in suitable habitat for 2187 m to 2083 m under 4.5 and 8.5 RCP in year 2070 (Table 3).

**Table 3:** The predicted uppermost and lowermost elevation range of finger millet suitable habitat in Nepal

Scenarios	Suitable elevation range	
	Minimum altitude (m)	Maximum altitude (m)
Current	96	2300
Future		
RCP 2.6		
Year 2050	89	2191
Year 2070	87	2175
RCP 4.5		
Year 2050	97	2151
Year 2070	96	2187
RCP6.0		
Year 2050	87	2186
Year 2070	94	2198
RCP 8.5		
Year 2050	96	2097
Year 2070	98	2083

## Discussion

The coverage of climatically suitable habitats was modeled for the first time in Nepal to delineate the potential suitable habitat of finger millet under current and future climate change scenarios.

Finger millet is relatively resistant to different stress conditions like high temperature, drought, and salinity of soil [30] and this crop follows photosynthetically efficient C4 pathway. Climate change projections reveal increased temperature by 4.7 °C in higher altitude areas of Nepal Himalayas within 2090 AD as well as monsoon rainfall likely to increase in erratic pattern by 2090 [8]. Our results clearly indicate that significant shrinkage of existing suitable area will occur with rising temperatures within 2050 by 3.9–10.3% and by 4.3–10.5% in 2070; however, there may be addition of suitable area by 4.6–5.6% within 2050 and 2.9–6.1% by 2070 under different RCPs in Nepal. No particular trend of loss and gain in the future were shown by the model, but net suitable area will be lost in 2050 and 2070 as compared to the current situation. Several climate related factors may play a role in the decline of suitable area of finger millet including drought, rise in maximum temperature, increasing erratic pattern of rainfall, and the shift of bioclimatic zones in different elevations in Nepal [31]. There was loss of agricultural land from Nepal during the period of 1971–2007 due to climate related catastrophes like droughts, flood, hailstorm, rain, strong winds, and cold wave. Around 38%of overall agricultural land loss was seen due to drought alone [32]. The projected suitable habitat losses mostly from mountains in western Nepal may be due to increase in drought in these regions because annual rainfall is the predictor variable with highest contribution in model performance in this study (Fig. 1).

The impact of climate change on suitable habitat would be mixed loss in suitable habitat in western region and some additional of suitable area in eastern and central parts are expected. A mixed impact of climate change on crops has been shown in banana and coffee in Nepal. With the change in climate, the suitable habitat of banana would increase, but that of coffee would worsen [33].

The addition of suitable area for finger millet in Nepal and downward shrinkage of highest elevation for cultivation from 2300 to 2080 m may be due to change in the average growing degree days (GDD) for finger millet from North to South [34] as temperature is one of the main limiting factors for crops, especially in temperate zones [35]. GDD is a measure of the heat a plant requires to mature and yield a successful crop. The predicted average rise in temperature of about 4.7 °C by 2090 [8] may not fulfill the daily air temperature at higher altitude during the growing season. Similarly, projected increases in erratic summer rainfall along with sharp slopes in the mountains are other limiting factors of the suitability of finger millet crop area from western parts of the country.

Changes in climatic conditions in the future will not only affect the crop distribution, but also its quality. Research conducted in the USA shows that the season-long high temperature stress reduces the chlorophyll index, seed number, grain yield and harvest index; however, only a few genotypes of finger millet show tolerance with high temperature [36]. Therefore, climate change will have at least double impact on finger millet production—first, shrinkage of suitable area, and second, the reduction in grain yield.

Siwalik and mid-hills between the elevation 500–1500 m have maximum suitable habitat for finger millet (Fig. 2a). Mid-hills in Gandaki, Lumbini, Sagarmatha regions of Nepal are the highest producers of finger millet [37], which is correctly reflected in the model results. However, from the visual estimation of model output in Fig. 2, the model means normally over estimatedthe suitable habitat in Mechi, Koshi, and Narayani regions and underestimated for Karnali regions (Fig. 2b–i). Nevertheless, high AUC, TSS and Kappa values suggest that the model has excellent level of accuracy (Table 1). The model accuracy has been verified with the comparison of elevation under current scenario and recorded elevation by other researchers. Based upon the literature, Kachorwa village (85 masl) of Bara district [38] is the lowest elevation and Borounse village (3130 masl) of Humla district [4] is the highest point of current cultivation in Nepal. From this model, the current moderate suitable (having probability in between 25 and 50%) climate for finger millet ranges between 75 and 3034 m asl, though most suitable area (having probability > 50%) lies in between 96 and 2300 m. This model prediction almost matched with the reality of current distribution of finger millet cultivation in Nepal.

## Conclusions

Maxent modeling for finger millet crop clearly defined the current suitable area for the cultivation and potential suitable areas in 2050 and 2070AD. The model revealed that 39.7% (58512.71 km^2^) area of Nepal is the most suitable habitat for finger millet, between 96 and 2300 m elevations, though only 4.5% of most suitable land is under cultivation at present cultivated land use of Nepal. Similarly, eastern and central parts of Nepal have more suitable areas than western parts. Our research clearly showed that the future climatic suitability of finger millet would shrink up to 10% under different RCPs from Nepal in 2070 and about 6% additional suitable area will emerge due to changing scenarios of climatic variables, by 2050 and 2070.The suitable habitat will decrease in the western mountain region (Karnali and Mahakali regions) as compared to other parts of the country. Although 10% suitable land would be lost due to climate change by 2070, another nearly 3–6% climatically suitable area would be available for finger millet cultivation, especially from mid land areas of the country. These findings help to develop the strategies to cultivate finger millet at climatically suitable area to fulfill the national demand.

## Materials and methods

### Crop species and location points

Finger millet (*Eleausine coracana)* was selected for modeling and its habitat suitability under climatic change scenarios. The diversity of finger millet (*Eleusine* sp.) is quite high, indicating that Nepal is one of the centers of origin [17], but a number of local landraces are either getting lost or on the verge of extinction. A total of 352 occurrence points of finger millet were collected from the field survey from 19 districts of central Nepal. In order to reduce the sampling biases in the location data of central Nepal, spatial filtering of occurrence points was used, employing fishnet option in Arc GIS10.3, which reduced the presence points to 101 for better performance of the model [18]. An additional 53 points were collected from the database of National Agriculture Genetic Resource Centre (NAGRC) and 250 points were collected from geo-referencing, principally based on the records of NAGRC that included spatial location names without geological coordinates. Thus, a total of 404 presence points of finger millet for the whole of Nepal were employed in Maxent model (Fig. 3).

### Bioclimatic variables

For current and future time periods (2050 and 2070 AD), all 19bioclimatic variables (bio30s) on 30-arc-sec resolution at ESRI grids format were downloaded from Worldclim dataset [19].The Community Climate System Model (CCSM_4_) [20] was followed under Representative Carbon Pathway of RCP 2.6, RCP4.5, RCP 6.0and RCP 8.5 for future (year 2050 and 2070) because CCSM_4_ is mostly used in South-Asian region for modeling. These data were statistically downscaled from Global Circulation Model (GCM) using Worldclim 1.4 at present baseline climate. The altitude, slope, and aspect were derived from the digital elevation data based on Shuttle Radar Topographic Mission (SRTM) at 90 m spatial resolution and were re-sampled to 30 arc second spatial resolutions to match with the resolution of climatic variables (Table 4). The raster data from the global scale was masked for Nepal. All RCPs (RCP’s 2.6, 4.5, 6.0 and 8.5) greenhouse concentration trajectories for two different time periods (2050 and 2070) were selected to determine the future habitat suitability of species. The lowest greenhouse concentration (GHG) pathway is RCPs 2.6 (aggressive mitigation/lowest emissions), while RCPs 4.5 and RCPs 6.0 are intermediate, and RCPs 8.5 (highest emission scenario) is maximum. GHG concentration pathways in which radioactive forcing (global energy imbalance) stabilizes 2.6 W/m^2^, 4.5 W/m^2^, 6.0 W/m^2^ and 8.5 W/m^2^, respectively, by 2100 [21, 22].

**Table 4:** Bioclimatic variables used for modelling habitat suitability of finger millet in Nepal

Code	Variables
nbio_1	Annual mean temperature
nbio_2	Mean diurnal range (mean of monthly (max temp—min temp))
nbio_3	Isothermality (Bio_2/Bio_7) (*100)
nbio_4	Temperature seasonality (standard deviation *100)
nbio_5	Max temperature of warmest month
nbio_6	Min temperature of coldest month
nbio_7	Temperature annual range (Bio_5–Bio_6)
nbio_8	Mean temperature of tettest quarter
nbio_9	Mean temperature of driest quarter
nbio_10	Mean temperature of warmest quarter
nbio_11	Mean temperature of coldest quarter
nbio_12	Annual precipitation
nbio_13	Precipitation of wettest month
nbio_14	Precipitation of driest month
nbio_15	Precipitation seasonality (coefficient of variation)
nbio_16	Precipitation of wettest quarter
nbio_17	Precipitation of driest quarter
nbio_18	Precipitation of warmest quarter
nbio_19	Precipitation of coldest quarter
nslope	Slope
naspect	Aspect
nsotar	soil

Source: worldclim.org

Among the 22 variables (19 bioclimatic variables, slope, aspect and altitudes), pair wise correlation and Variation Inflation Factors (VIFs) were performed by using statistical software R [23] to test the multi co-linearity among the environmental variables. The highly correlated bio-variables with a Pearson correlation (r. ≤ ±0.80) and VIF > 5 were omitted in order to reduce the effect of multi-co-linearity among the variables, which enables over fitting of the model [24, 25].

The remaining nine variables viz. aspect, slope, mean diurnal range (mean of monthly{max temp-min temp}), isothermality (Bio_2/Bio_7) (*100),temperature seasonality (standard deviation *100), mean temperature of driest quarter, annual precipitation, precipitation of coldest quarter and soil were used to model the habitat suitability of finger millet in current and future climatic conditions. All the bioclimatic data sets were converted into ASCII files in Arc GIS 10.3 to make the acceptable format for Maxent software. The same process was repeated to produce the projected maps in all future scenarios for the year 2050 and 2070 considering the soil condition to remain constant in the future under RCP2.6, RCP4.5, RCP6.0 and RCP8.5.

### Modeling by maxent

The open source Maxent software (Maxent 3.4.1) was used to quantify (model) the current and future habitat suitability of finger millet cultivation area in Nepal. Maxent is a machine learning method that estimates the probability distribution of a species occurrence based on environmental conditions of a location in which the species is found by calculating the distribution of maximum entropy i.e. the most spread out distribution in space for a given set of constraints [16]. In this study, the Maxent software was modeled by applying the following parameters—25% random test percentage, i.e. 75% of presence points data were used for training and the remaining 25% to test the predictive ability of Maxent model, 1 regularization multiplier, 10,000 maximum numbers of background points, 10 replicates, subsample replicate type, 5000 maximum iterations, 0.00001 convergence thresholds. The file format was set for logistic output, which provides predicted probabilities in between 0 and 1 and background prediction.

### Model validation

The Area Under Receiver-operating Characteristic Curve (AUC), Kohan’s Kappa and true skill statistic (TSS) were used for model evaluation [26].

The presence dataset was divided 75 percent into training data, which were used to build a model, and the remaining 25% for test data was used to test the model performance [27]. The values of AUC range from 0.5 to 1.0, with 0.5 indicating no (random) fit to the data, 1.0 indicating perfect model performance, 0.7–0.79 reasonable, 0.8–0.89, excellent, and values > 0.9 indicating high performance [28]. The TSS account for both sensitivity and specificity and its values lie within + 1 to − 1, where + 1 indicates the perfect model performance [26]. To assess the importance of used variables to the model, Jackknife procedure was used.

The final potential distribution map with values 0 to1 were grouped into two classes viz. 0.00 to 0.5 and 0.5 to 1.0. We considered pixels with or more than 0.50 value to consider areas that depict at least 50% probability of species cultivation suitability [29]. The stability gain and loss of predicted suitable area was calculated for future in each RCP scenario through Arc GIS 10.3. To calculate stability, gain and loss on predicted area, the final output map of Maxent was binarized (i.e. presence/absence) by using lowest value of 10 percentile training presence Logistic threshold of Maxent results. The stable, gain and loss of suitable area map was determined by using intersect and symmetrical analysis tools in Arc GIS 10.3.

## Data Availability

The datasets of this study will be available from corresponding authors on genuine request.

## Abbreviations

RCP: Representative concentration pathways
GDP: Gross domestic products
ReCM: Regional circulation model
SDM: Species distribution modelling
AUC: Area under receiver-operating characteristic curve
TSS: True skill statistic
NAGRC: National agriculture genetic resource centre
CCSM_4_: Community climate system model
GHG: Green house gas
VIF: Variation inflation factors
Maxent: Maximum entropy
